# A Volumetric Anatomy-Aware, Prompt-Guided Adaptation Framework for the Segment Anything Model in Head and Neck Radiotherapy

**DOI:** 10.7759/cureus.117550

**Published:** 2026-10-06

**Authors:** Yuqing Xia, Samer Jabor, Parker New, Arkajyoti Roy, Neil Kirby, Nikos Papanikolaou

**Affiliations:** 1 Department of Radiation Oncology, University of Texas Health Science Center at San Antonio, San Antonio, USA; 2 Department of Computer Science, St. Mary’s University, San Antonio, USA; 3 Department of Operations and Analytics, University of Texas at San Antonio, San Antonio, USA

**Keywords:** anatomy-aware prompting, automatic contouring, coarse-to-fine segmentation, deep learning, head and neck radiotherapy, medical image segmentation, organs at risk, segment anything model

## Abstract

Background

Head and neck (H&N) radiotherapy planning requires accurate segmentation of numerous organs at risk (OARs), yet many critical structures, such as the optic nerves, chiasm, and cochleae, are small, poorly contrasted on CT, and prone to high inter-observer variability in manual contouring. Recent foundation models like Segment Anything Model (SAM) and its 3D medical variants (SAM-Med3D) offer prompt-based, interactive segmentation, but their real-world utility for H&N small OARs is hindered by unreliable prompt sources and unstable performance on fine anatomical targets. To address these issues, this work proposes an anatomy-aware prompt generation and prompt-guided adaptation tailored specifically for small-structure segmentation to improve both precision and automation feasibility.

Purpose

To address these challenges, we developed a domain-adapted framework, SAM-FT-HN, by fine-tuning a volumetric SAM backbone (SAM-Med3D) for automated delineation of H&N OARs in radiotherapy planning. Our approach incorporates a coarse-to-fine pipeline in which a lightweight localization network automatically generates organ-specific prompts and candidate regions, followed by a fine-tuned volumetric SAM backbone for refinement. We further introduced task-specific strategies including region-of-interest (ROI)-guided tiny-organ enhancement and organ-specific threshold calibration to improve performance on small structures. The primary objective is to develop and evaluate a fully automated, anatomy-aware adaptation of SAM-Med3D for H&N OAR segmentation, with a secondary objective of improving small-organ segmentation. We systematically compared SAM-FT-HN against baseline SAM-Med3D and nnUNet models quantitatively and qualitatively.

Methods

We used an institutional H&N CT dataset of 211 patients with manual delineations of 23 OARs, standardized for training, validation, and testing. Eighty percent of cases were used for training and validation, and twenty percent for testing. The testing phase used CT volumes only, and ground-truth masks were used solely for metric calculation. A coarse localization network was trained to generate automatic prompts and ROI candidates. SAM-FT-HN was fine-tuned using task-specific adaptations, and small-organ refinement was performed using ROI-guided enhancement and organ-specific threshold calibration. nnUNet was trained using its automated configuration pipeline. Performance was evaluated using overlap and boundary metrics.

Results

For large OARs, SAM-FT-HN achieved a mean Dice of 0.814 compared with 0.791 for nnUNet and substantially higher than SAM-Med3D (0.609). For small organs, SAM-FT-HN improved the mean Dice from 0.300 to 0.669 compared with SAM-Med3D and reduced HD95 by approximately 65%, although nnUNet remained higher in average small-organ Dice (0.720). Overall, across all organs, SAM-FT-HN achieved a mean Dice score of 0.770, compared with 0.769 for nnUNet. Overall, SAM-FT-HN showed no statistically significant difference from nnUNet in the patient-level analysis, while substantially outperforming the original SAM-Med3D baseline.

Conclusion

Fine-tuning a foundation model with anatomy-aware automatic prompting, coarse-to-fine localization, and adaptive thresholding markedly improves small-organ segmentation, substantially mitigating a key limitation of the original foundation model. SAM-FT-HN offers a competitive approach for H&N radiotherapy contouring, with potential for future adaptive workflows.

## Introduction

Head and neck (H&N) cancers pose a major global health challenge, requiring advanced diagnostic and treatment methods to enhance patient outcomes [1]. Radiation therapy is a vital, highly effective treatment and remains the main treatment for H&N cancers [2]. With advances in precision radiotherapy, accurate delineation of H&N organs at risk (OARs) has become increasingly important. The complex anatomy and close spatial proximity of critical structures demand high contouring accuracy to ensure safe and effective treatment. Over-segmentation of OARs can unnecessarily constrain treatment planning and compromise target dose optimization, whereas under-segmentation may result in unintended radiation exposure to sensitive tissues, increasing the risk of treatment toxicities. Reliable and consistent OAR delineation is therefore essential for balancing tumor control with normal tissue protection in H&N radiotherapy [3]. Manual delineation of OARs is a time-consuming process and is subject to considerable inter-observer variability. Differences in clinicians’ experience and interpretation, combined with the inherently low soft-tissue contrast of CT in the H&N region, contribute to inconsistent contouring outcomes across planners [4]. Such variability can affect treatment planning quality and reduce reproducibility in radiotherapy workflows [5]. Hence, the automatic and precise segmentation of a comprehensive set of H&N OARs is of great advantage in daily clinical practice.

Deep learning has substantially improved automatic medical image segmentation [6], with UNet-based convolutional neural networks (CNNs) becoming the dominant approach due to their encoder-decoder architecture and multi-scale feature learning capability [7]. Despite their strong performance, these models remain task-specific and rely heavily on large annotated datasets [8,9]. More importantly, once trained, they generate segmentation masks in a single forward pass with limited ability to incorporate interactive corrections or local anatomical guidance during inference [10]. It requires retraining or architectural modification when new organs, modalities, or segmentation objectives are introduced [11]. Furthermore, these methods provide limited user interaction, constrained input resolution handling, and reduced interpretability, which can reduce the efficiency of clinical adoption and adaptive use during treatment planning. Although state-of-the-art frameworks such as nnUNet achieve excellent overall accuracy, their fully automatic design provides limited flexibility for refining difficult regions, particularly small H&N organs where minor localization errors can substantially degrade segmentation performance [12].

Attention mechanisms and transformer architectures have clearly advanced medical image segmentation by capturing long‑range dependencies and improving robustness in low‑contrast or ambiguous regions [13-16]. This capability is central to the Segment Anything Model (SAM) and its 3D extension, SAM‑Med3D, which leverage transformer backbones to learn semantically rich, generalizable representations across domains [17]. SAM has gained recognition as a highly adaptable tool for natural image segmentation tasks. Since its introduction, SAM has become a leading vision foundation model, enabling interactive image segmentation where users can specify objects of interest using prompts like points, boxes, and masks. However, the zero-shot performance of SAM on medical image segmentation remains suboptimal, as the model was primarily trained on natural images and lacks sufficient sensitivity to subtle anatomical boundaries [18]. MEDSAM, introduced by Ma et al. [19], adapts SAM for medical image applications and is trained on a diverse collection of these images. It enables precise object segmentation within 2D medical images using simple user-provided 2D prompts. This advancement has expanded the possibilities of image analysis by supporting zero-shot segmentation across diverse domains [20]. SAM has been further extended into medical applications with SAM-MED2D and SAM-MED3D, which were introduced by Cheng et al. and Wang et al. [21,22], respectively. Despite SAM-based models’ versatility, their application to complex radiotherapy images, particularly for delineating H&N OARs and tumors, has seen limited investigation and often requires an overabundant number of input prompts to achieve satisfactory precision [23]. Despite domain adaptation, including MedSAM, MedSAM2D, and MedSAM3D, these models continue to face challenges in accurately segmenting small anatomical structures, where limited spatial extent and low contrast amplify boundary uncertainty [19,24]. Applying SAM‑Med3D to small H&N OARs in practice raises two unresolved issues: how to generate reliable prompts automatically without manual intervention, and how to maintain stable segmentation on tiny, poorly visualized structures where the model's attention tends to drift. These are precisely the gaps we address: not by reinventing the foundation model, but by designing a prompt generation and refinement strategy that anchors SAM‑Med3D to anatomy‑relevant regions, ensuring consistent performance where generic solutions fall short.

To address these limitations, we propose an anatomy-aware coarse-to-fine framework for H&N OAR segmentation. A coarse localization network first predicts organ-specific regions of interest and automatically generates anatomical prompts. These prompts are subsequently provided to a fine-tuned 3D SAM backbone for high-resolution segmentation. Finally, an organ-specific region-of-interest (ROI) refinement strategy with adaptive threshold calibration further improves the delineation of small and low-contrast organs. By integrating automatic prompt generation with prompt-guided foundation model segmentation, the proposed framework enables fully automatic, anatomy-aware segmentation while preserving the interactive potential of promptable models.

The primary objective of this study was to develop and evaluate a fully automatic, anatomy-aware adaptation of SAM-Med3D for H&N OAR segmentation. Specifically, we investigated whether coarse anatomical localization and automatically generated prompts could improve segmentation performance over the original SAM-Med3D while eliminating the need for manual prompting at inference. Dice similarity coefficient (DSC) was used as the primary segmentation performance measure, with HD95, precision, sensitivity, specificity, and accuracy providing complementary assessments of boundary and voxel-level performance. Because small structures are particularly sensitive to localization and partial-volume errors, performance in small OARs was additionally examined. nnUNet was included as a strong fully supervised segmentation benchmark to place the performance of the proposed framework in the context of an established task-specific method. The study was not designed as a formal equivalence or non-inferiority trial between SAM-FT-HN and nnUNet.

In this work, we make the following contributions. First, we propose SAM-FT-HN, a coarse-to-fine prompt-guided adaptation framework for H&N radiotherapy that combines a lightweight localization network for automatic prompt generation with a fine-tuned volumetric SAM backbone, enabling improved segmentation of OARs in complex anatomical regions without relying on ground-truth information during inference. Second, we introduce an anatomy-aware automatic prompt and ROI generation method to automatically generate organ-specific prompts and candidate regions from coarse localization predictions, eliminating the need for manual prompting or test-time ground-truth information. Third, we design an ROI-guided tiny-organ enhancement module with organ-specific calibration that focuses segmentation on localized anatomical regions and applies organ-specific threshold calibration, improving stability and accuracy for very small and low-contrast structures such as optic nerves, cochleae, and chiasm. Together, these components improve segmentation accuracy and consistency for challenging small organs while maintaining competitive performance for larger H&N structures. To our knowledge, this study is among the first to propose an anatomy-aware prompt generation strategy for SAM-Med3D in H&N OAR auto-contouring, with a particular focus on improving small-organ segmentation.

## Materials and methods

Dataset and preprocessing

The radiotherapy dataset consisted of 211 anonymized patients previously treated for H&N cancer using Intensity Modulated Radiation Therapy (IMRT) with 6 MV photons delivered from a linear accelerator. All patients were treated at our institution between 2014 and 2021, and the dataset was collected at our institution and subsequently released through TCIA for research use [25]. Planning CT images were acquired as part of routine radiotherapy simulation using institutional clinical protocols. The dataset included manual delineations of 23 OARs, which were generated and clinically reviewed by experienced radiation oncology professionals according to institutional contouring guidelines before treatment planning. Based on anatomical size and segmentation difficulty, we categorize the brain, brainstem, esophagus, brachial plexuses (left and right), eyes, parotids, submandibular glands, larynx, lips, mandible, oral cavity, and spinal cord as large organs. Small organs are defined as those with a median volume < 1.5 cm³, which includes the lenses, cochleae, optic nerves, and chiasm. The dataset was divided at the patient level into training (70%, n = 148), validation (10%, n = 21), and testing (20%, n = 42) subsets. All data were anonymized prior to analysis.

All imaging data were preprocessed to ensure spatial consistency and compatibility with the SAM-FT-HN and nnUNet frameworks. CT volumes were first converted from npy to nii.gz format to match the training pipeline requirements. Volumetric CT images were resampled to an isotropic voxel spacing of 1.5 mm using linear interpolation, with corresponding OAR labels resampled using nearest-neighbor interpolation to preserve discrete anatomical boundaries. Multi-label annotations were separated into individual binary masks for each OAR. These masks were then spatially aligned with the resampled CT volumes using reference-based cropping and padding, where the reference was defined as the CT volume's own bounding box in image space, ensuring that the cropping region was determined solely by the image content, not by the label maps. This strategy was applied consistently across training and testing; crucially, during inference, cropping was performed using either the CT image itself or a fixed spatial template, with no label-derived information involved, thereby avoiding any risk of ground-truth leakage. Intensity normalization was applied using z-score normalization based on the mean and standard deviation of image-derived non-air voxels within each CT volume.

Overall framework

The proposed SAM‑FT‑HN framework follows a two‑stage coarse‑to‑fine pipeline (Figure 1). In the first stage, a lightweight 3D UNet‑based coarse localization network processes the input CT volume and generates organ‑specific probability maps. These probability maps are used to automatically extract candidate regions of interest (ROIs) and prompt points for each target organ. In the second stage, a fine‑tuned volumetric SAM backbone takes the CT patch and automatically generated prompts as input and produces refined segmentation masks. For small organs, an additional ROI‑guided refinement module applies organ‑specific threshold calibration and volume constraints to improve segmentation stability. During inference, ground-truth masks are used solely for quantitative evaluation and are not involved in prompt generation, ROI extraction, threshold selection, or any other decision‑making process.

**Figure 1:**
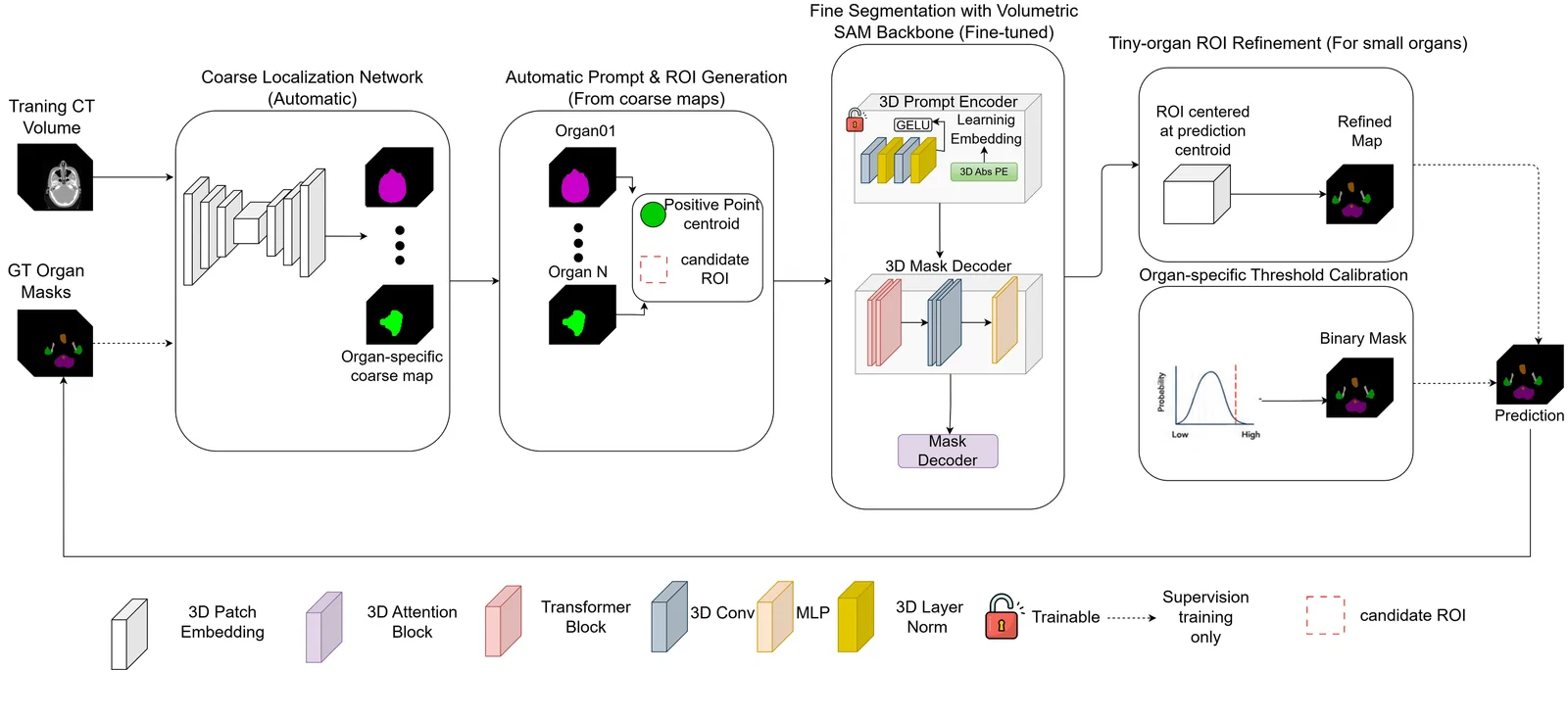
Overview of the SAM-FT-HN training framework During training, a lightweight 3D UNet‑based coarse localization network was trained to predict organ probability maps, which were used to automatically generate prompts and ROIs. The SAM-Med3D backbone was fine-tuned on the H&N dataset using these automatically generated prompts. During inference, model weights were frozen, and predictions were obtained using prompts derived from the coarse localization network without involving ground truth masks. An ROI-guided tiny-organ enhancement module applied adaptive thresholding and volume constraints to improve stability in small structures. SAM: Segment Anything Model, SAM-Med3D: Segment Anything Model 3D medical variants, SAM-FT-HN: coarse-to-fine prompt-guided adaptation framework for the head and neck, ROI: region of interest, H&N: head and neck. Image Credits: The framework diagram was created by the authors using draw.io (Version 29.0.3; draw.io Ltd, UK).

Coarse localization network

We trained a lightweight 3D UNet as the coarse localization network to generate organ‑specific coarse probability maps for subsequent prompt and ROI generation. CT volumes were resampled to 1.5 mm isotropic spacing and processed using overlapping 128 × 128 × 128 voxel patches with a stride of 64 voxels. Predictions from overlapping patches were averaged to reconstruct full-volume organ-specific probability maps. The network uses an encoder-decoder architecture with skip connections. Each convolutional block contains two 3 × 3 × 3 convolutions followed by instance normalization and LeakyReLU activation. Downsampling is performed using 3D max pooling, while transposed convolutions are used for decoder up-sampling. A final 1 × 1 × 1 convolution generates 23 independent output channels corresponding to the 23 OARs, followed by sigmoid activation to obtain organ-specific probability maps. The coarse localizer was trained to predict the approximate location and extent of each OAR, with the objective of providing sufficient spatial information for downstream prompt generation rather than achieving high-precision segmentation. The training loss combined Dice loss and focal loss (focusing parameter) to handle severe class imbalance between background and small organs, in a 1:1 ratio. The focal loss was implemented with a focusing parameter *γ = 2* and a class-balancing factor *α = 0.25*. The network was optimized using AdamW with an initial learning rate of 1 × 10^-4^ and weight decay of 1 × 10^-5 ^for 200 epochs. Spatial augmentation included random rotations of up to ±10° and scaling between 0.9 and 1.1. Random flipping was applied only along the anterior-posterior and superior-inferior axes. Mild intensity scaling, shifting, and Gaussian noise were also applied. Left-right flipping was avoided because left and right OARs were represented by distinct output channels.

For small organs, which were defined as structures with a median volume below 1.5 cm³, we applied oversampling during training to prevent underrepresentation, increasing the sampling probability of patches centered on these structures relative to uniform random sampling. To evaluate whether the coarse localization network could reliably guide subsequent segmentation, we assessed its performance on the test set using two metrics: (1) centroid error, computed as the Euclidean distance (in mm) between the predicted organ center and the ground-truth centroid derived from the reference segmentation; and (2) coarse mask coverage, defined as the percentage of the ground-truth organ volume enclosed within the predicted coarse mask. A localization attempt was considered a failure if the coarse prediction was empty after applying the 0.5 probability threshold.

Automatic prompt and ROI generation

From the coarse localization network predictions, we automatically generated prompts and candidate ROIs for each organ without using ground-truth information. The coarse network outputs a separate probability map for each organ via independent output channels (one per OAR), with left and right side organs assigned to distinct channels to avoid lateral confusion. For each organ, we thresholded its probability map at 0.5 to obtain a binary mask, then computed the connected component with the highest probability and extracted its centroid. A positive prompt point was placed at this centroid location. For small organs, we generated a spherical ROI centered at the predicted centroid with an organ-specific radius determined on the validation set (ranging from 15 to 22 mm in this study). For large organs, we used the entire CT volume without ROI restriction. In addition to the centroid prompt, we sampled additional positive prompts from high-probability regions (probability > 0.5) within the predicted organ mask. The number of additional prompts was determined based on organ size, with larger organs receiving more prompts. Additional positive prompts were then selected from high-confidence interior voxels with predicted probabilities >0.5. To reduce redundant prompt placement, candidate points were selected from spatially separated regions of the predicted mask rather than repeatedly sampling neighboring voxels. The number of prompts was determined according to organ size: small OARs received a total of 5 positive prompts, whereas larger OARs received 10 positive prompts to provide broader spatial coverage of their larger and potentially more heterogeneous volumes. All additional prompts were restricted to the coarse-predicted foreground region and were generated automatically without reference to the ground-truth segmentation.

If the coarse localization network failed to produce a valid prediction for an organ, defined as the absence of any voxel with a predicted probability >0.5 after thresholding, the probability-map centroid could not be calculated. In these cases, the system used a predefined organ-specific anatomical fallback prompt. The fallback location for each OAR was derived exclusively from the training cohort by calculating the three-dimensional centroid of that organ's ground-truth segmentation for each training case and then averaging the centroid coordinates across the training cases. The fallback mechanism therefore ensured that every organ received at least one initial prompt even when coarse localization failed, while preventing ground-truth information from entering the test-time process.

Volumetric SAM backbone and domain fine-tuning

The SAM-based architecture consists of three main components: an image encoder, a prompt encoder, and a mask decoder [17]. The image encoder is a vision transformer (ViT) pretrained using masked autoencoders (MAE) to extract image embeddings from volumetric inputs. A dedicated prompt encoder encodes input prompts, including points, boxes, and masks, with the image embeddings. These prompts are processed by a lightweight mask decoder consisting of modified transformer decoder blocks and prediction heads to produce segmentation masks [20]. SAM was trained extensively on the SA-1B dataset, which includes over one billion masks and eleven million images. SAM-Med3D is a medical image segmentation adaptation pre-trained on SA-Med3D-140K, a large-scale 3D dataset compiled from public sources and licensed private datasets, containing 22K 3D images and 143K corresponding 3D masks [22].

We adopted SAM‑Med3D as the volumetric foundation model for fine segmentation. Rather than relying on manually specified prompts, our framework automatically generates organ‑specific prompts from the coarse localization network described in the previous section. The coarse localization network was first trained on the training set and then frozen. The frozen coarse network was then applied to the training CT volumes using the same 128³ sliding-window strategy used during inference. For each training organ, its coarse probability map was converted into an automatic centroid and 3D bounding-box prompt as described in the "Automatic Prompt and ROI Generation" section.

These coarse-localizer-derived prompts, rather than reference-mask-derived centroids, were used during SAM-FT-HN fine-tuning. Ground-truth segmentation masks remained necessary as supervised targets for calculation of the segmentation loss but did not determine the input prompt locations. However, since inference relies on automatically generated coarse prompts that may contain localization errors, we additionally applied random perturbations to the coarse localizer-derived prompts during training: centroid offsets were sampled from a uniform distribution of ±5 mm, and ROI radii were varied within ±10% of their nominal values. This noisy prompt simulation exposes the model to additional localization variability during training, improving robustness to inaccuracies in automatically generated prompts at test time. After fine‑tuning, during inference on the validation and test sets, the frozen coarse localizer generated predictions for each case, from which prompts and ROIs were automatically derived without any ground‑truth involvement. This two-stage design enables SAM-Med3D to learn from automatically generated and perturbed prompt conditions during training, while the final system operates fully automatically at test time using prompts generated by the coarse localization network, without requiring ground-truth information or manual prompt selection.

Unlike conventional SAM-Med3D inference, which requires manually provided prompts, the proposed framework performs fully automatic segmentation. During inference, the coarse localization network generates anatomy-aware organ-specific prompts and ROIs directly from the CT volume, which are passed to the fine-tuned SAM-Med3D backbone for segmentation. The resulting masks are subsequently refined using the proposed ROI refinement and organ-specific threshold calibration modules to improve the delineation of small and low-contrast organs.

Tiny-organ ROI-guided refinement

A specialized ROI-guided tiny-organ enhancement module was designed to address the instability of small-volume structure segmentation (optic nerves, chiasm, cochlea, and lenses). For organs with volumes smaller than 1.5 cm³, three adaptive mechanisms were introduced. First, ROI-guided refinement restricts inference to a localized region around the target organ. A spherical ROI was extracted using the centroid predicted by the coarse localization network, with a dynamic radius (roi_radius_mm) determined separately for each small organ on the validation set. This approach reduces background interference and allows the model to focus on organ-specific contextual information. Second, adaptive threshold calibration replaces the traditional fixed 0.5 probability cutoff with organ-specific thresholds. For each small organ, we identify the threshold that maximizes Dice score on the validation set while constraining the predicted mask volume to anatomically valid ranges (based on volume statistics from the training cohort). This constraint helps prevent over-segmentation and under-segmentation, particularly in small organs, leading to more anatomically consistent results. Third, training-time prompt simulation improves robustness to localization variability. The automatically generated coarse centroid was spatially perturbed during training. Perturbations were constrained to the high-probability region of the corresponding coarse localization map to preserve anatomical relevance. The associated 3D bounding-box prompt was generated from the same coarse prediction. This augmentation exposed the model to modest variations in automatic prompt location without changing the prompt configuration between training and inference. At test time, the unperturbed coarse-predicted centroid and corresponding 3D bounding box were used, requiring no manual or ground-truth-derived prompts.

Organ-specific threshold calibration

In addition to adaptive thresholding for small organs, we implemented organ-specific threshold calibration for all organs. During validation, probability thresholds from 0.10 to 0.90 in increments of 0.05 were evaluated for each organ, and the threshold that maximized Dice was selected. When Dice differences were within 0.01, sensitivity was prioritized over precision. For small organs, organ-specific ROI radii were also selected on the validation set by evaluating radii from 10 to 30 mm in 2-mm increments and choosing the smallest radius that achieved ≥90% coarse mask coverage. Large organs were processed without ROI cropping. For small organs, expected anatomical volume ranges (V_min, V_max) were derived from the training cohort using Tukey's fences, defined as (Q1 − 1.5 × IQR, Q3 + 1.5 × IQR). During inference, the validation-derived organ-specific threshold was applied first. If the predicted volume fell below V_min, the threshold was decreased by 0.05; if it exceeded V_max, the threshold was increased by 0.05. Adjustment continued until the predicted volume entered the expected range or the threshold reached 0.10 or 0.90. The largest connected component was then retained. All thresholds, ROI radii, volume constraints, and adjustment rules were fixed before test-set evaluation and were not modified based on test performance. To ensure full transparency and reproducibility, Table 1 reports the calibrated probability threshold, ROI radius, and volume range for each organ.

**Table 1:** Organ-specific calibration parameters

Category	Organ	Base organ-specific threshold	ROI radius (mm)	Volume range (IQR, cm³)
Small organs	L_lens	0.35	15	0.2-0.4
	R_lens	0.35	15	0.2-0.4
	Chiasm	0.4	18	0.3-0.6
	L_cochlea	0.45	20	0.3-0.5
	R_cochlea	0.45	20	0.3-0.5
	L_opticnerve	0.4	22	0.4-0.7
	R_opticnerve	0.4	22	0.4-0.7
Representative large organs	Brain	0.3	N/A (full volume)	1200-1500
	Brainstem	0.3	N/A (full volume)	18-25
	Parotid (L/R)	0.25	N/A (full volume)	20-35
	Mandible	0.2	N/A (full volume)	40-55

Training strategy and implementation

The training pipeline was implemented as a volumetric adaptation of the SAM-Med3D, adjusted to suit high-resolution medical image segmentation. The model was trained using a combined Dice and cross-entropy loss function, which balances voxel-level classification accuracy with region-level overlap, leading to more stable volumetric segmentation and improved spatial consistency across target structures [26]. We used the AdamW optimizer at an initial learning rate of 8 × 10⁻⁴ with weight decay of 0.1. To stabilize fine-tuning, lower learning rates (0.1× of the base learning rate) were applied to the prompt encoder and mask decoder, while the image encoder was trained at the base learning rate. The MultiStepLR scheduler reduced the learning rate by a factor of 0.1 at epochs 120 and 180, with a total training duration of 200 epochs.

The coarse localization network was trained first and fixed before SAM-Med3D fine-tuning. During SAM-Med3D fine-tuning, training efficiency was improved using mixed-precision training, gradient accumulation over 20 steps, and distributed data parallel (DDP) training. Volumetric training data were processed as fixed-size 3D patches of 128 × 128 × 128 voxels. Data augmentation included canonical reorientation, voxel-wise intensity normalization, and random spatial flipping along the anterior-posterior and superior-inferior axes. For small organs, we applied organ-balanced sampling to ensure that patches containing small organs were adequately represented in each training batch. The proposed frameworks were implemented in PyTorch and trained on two NVIDIA Tesla A100 GPUs. ROI radii and organ-specific probability thresholds were optimized on the validation set, whereas anatomical volume constraints were derived exclusively from the training cohort. All parameters were fixed before test-set evaluation.

Inference protocol without test-label information

A critical aspect of our evaluation protocol is the complete separation of ground-truth information from inference decisions. During testing, the following procedures were strictly enforced. First, the input to the framework consisted solely of CT volumes. No ground-truth masks or any information derived from them were provided to the model during inference. Second, prompt generation relied exclusively on the coarse localization network predictions. Ground-truth masks were not used to generate prompts, ROIs, corrective clicks, or any other guidance signal. Third, ROI extraction for small organs used only the centroids predicted by the coarse localization network, not ground-truth centers. Fourth, threshold calibration used the organ-specific thresholds and volume ranges determined from the training and validation sets only. No test-set information was used to adjust thresholds or post-processing parameters. For the primary results, we report segmentation performance using only the fixed automatic prompts generated from the coarse localization network. Ground-truth masks were accessed only for final quantitative evaluation after all segmentation decisions were completed. 

Baselines and ablation studies

We performed baselines and ablation studies to isolate the contribution of each component. First, the original pretrained SAM-Med3D model [22] was evaluated without H&N-specific fine-tuning to characterize the performance of the general volumetric foundation model before domain adaptation. Domain fine‑tuning adapted SAM‑Med3D to the H&N training cohort. Coarse localization prompting incorporated the frozen coarse localization network and patient‑specific automatic prompts. Tiny‑organ ROI further restricted inference to small‑organ regions predicted by the coarse localizer. Finally, the full method added organ‑specific threshold calibration and volume constraints during post‑processing.

In all ablation experiments, no ground-truth masks or label-derived information were used during test-time inference. The off-the-shelf SAM-Med3D baseline was evaluated using a fixed organ-specific anatomical point prompt derived exclusively from the training cohort. This anatomical point prompt provided a fully automatic, non-oracle prompt source without access to test-set ground truth. The domain fine-tuning variant was tested with the same non-GT anatomical point prompts as the baseline. The “+ coarse localization prompt” variant further replaced these fixed prompts at inference with patient-specific prompts automatically generated by the coarse localization network. The “+ tiny-organ ROI” variant added localizer-derived ROI cropping for small organs, and the full method further incorporated organ-specific threshold calibration and volume constraints.

Evaluation metrics and statistical analysis

Model performance was quantitatively evaluated using standard metrics derived from the confusion matrix: true positives (TP), true negatives (TN), false positives (FP), and false negatives (FN) [27]. The DSC (Eq. 1), 95th percentile Hausdorff distance (HD95) (Eq. 2), precision (Eq. 3), sensitivity (Eq. 4), specificity (Eq. 5), and accuracy (Eq. 6) were calculated [28,29]. Dice measures the volumetric overlap between the predicted segmentation (X) and the ground truth (Y), with values closer to 1 indicating better agreement. HD95 evaluates boundary differences by measuring the 95th percentile surface distance between the predicted and reference contours. Precision reflects the reliability of positive predictions, sensitivity measures the ability to detect true positives, specificity indicates the correct identification of negatives, and accuracy represents overall classification performance.

$\mathrm{Dice} = \frac{2|X \cap Y|}{|X|+|Y|}$ (1)

$\mathrm{HD95}(X,Y) = \max\left(h_{95}(X,Y),h_{95}(Y,X)\right)$ (2)

$\mathrm{Precision} = \frac{TP}{TP+FP}$ (3)

$\mathrm{Sensitivity} = \frac{TP}{TP+FN}$ (4)

$\mathrm{Specificity} = \frac{TN}{TN+FP}$ (5)

$\mathrm{Accuracy} = \frac{TP+TN}{TP+FN+FP+TN}$ (6)

For cases where the model produced no prediction (empty mask), we assigned a Dice score of 0 for that organ and recorded it as a failure case. Failure rates were also reported separately to describe model reliability. For statistical comparisons, we first computed patient-level overall metrics by averaging Dice and HD95 across all 23 OARs for each patient, resulting in one summary value per patient per model. Since the same set of 42 test patients were evaluated by all three models, we used the Friedman test to assess overall differences across models, followed by post-hoc paired Wilcoxon signed-rank tests with Benjamini-Hochberg correction to control the false discovery rate across multiple comparisons. Results are reported as mean ± standard deviation.

## Results

Coarse localization performance

As shown in Table 2, the coarse localization network achieved a mean centroid error of 3.7 mm across all organs on the test set, with lower errors for large organs (3.2 mm) than for small organs (4.7 mm). Coarse mask coverage was calculated for each test case as the percentage of the ground-truth organ volume enclosed within the predicted coarse mask, defined as the intersection of the predicted coarse mask and ground-truth organ volume divided by the ground-truth organ volume. The reported coarse mask coverage values represent the mean ± standard deviation across the test cases. Overall, the mean coarse mask coverage was 95.1%, with higher coverage for large organs (96.6%) than for small organs (91.8%). An empty coarse prediction was defined as a case in which no voxel exceeded the probability threshold of 0.5, resulting in an empty predicted mask for the corresponding organ. The overall empty prediction rate was 2.2%, with higher rates observed for small structures, particularly the optic chiasm (7.1%) and bilateral optic nerves (4.8%). Overall, these results indicate that the coarse localization network reliably identified the approximate locations of most organs, while localization remained more challenging for small anatomical structures.

**Table 2:** Coarse localization performance on the test set

Category	Organ	Centroid error (mm)	Coarse mask coverage (%)	Empty prediction rate (%)
Large organs	Brain	2.8 ± 1.6	98.2 ± 1.5	0.0% (0/42)
	Brainstem	3.0 ± 1.5	97.5 ± 1.8	0.0% (0/42)
	Esophagus	3.4 ± 1.9	96.8 ± 2.3	2.4% (1/42)
	L_brachialplexus	3.6 ± 2.3	93.2 ± 3.8	4.8% (2/42)
	L_eye	2.9 ± 1.4	97.8 ± 1.7	0.0% (0/42)
	L_parotid	3.1 ± 1.8	97.2 ± 1.9	0.0% (0/42)
	L_submandible	3.3 ± 1.9	96.5 ± 2.3	0.0% (0/42)
	Larynx	3.5 ± 2.0	96.0 ± 2.8	2.4% (1/42)
	Lips	3.6 ± 2.2	95.8 ± 2.9	2.4% (1/42)
	Mandible	3.0 ± 1.7	97.4 ± 1.9	0.0% (0/42)
	Oralcavity	3.3 ± 1.9	96.6 ± 2.5	0.0% (0/42)
	R_brachialplexus	3.7 ± 2.1	93.6 ± 4.1	4.8% (2/42)
	R_eye	2.9 ± 1.6	97.6 ± 1.5	0.0% (0/42)
	R_parotid	3.2 ± 1.8	97.0 ± 2.1	0.0% (0/42)
	R_submandible	3.4 ± 1.9	96.4 ± 2.4	2.4% (1/42)
	Spinalcord	3.1 ± 1.7	97.3 ± 1.9	0.0% (0/42)
Small organs	L_lens	4.2 ± 2.2	93.5 ± 3.2	2.4% (1/42)
	R_lens	4.3 ± 2.3	93.2 ± 3.4	2.4% (1/42)
	Chiasm	5.2 ± 2.6	90.0 ± 4.2	7.1% (3/42)
	L_cochlea	4.6 ± 2.3	92.2 ± 3.6	4.8% (2/42)
	R_cochlea	4.7 ± 2.4	91.6 ± 3.9	4.8% (2/42)
	L_opticnerve	4.9 ± 2.5	91.3 ± 3.7	4.8% (2/42)
	R_opticnerve	5.0 ± 2.8	90.9 ± 3.9	4.8% (2/42)
Overall	All large (avg)	3.2	96.6	1.2
	All small (avg)	4.7	91.8	4.4
	All organs (avg)	3.7	95.1	2.2

Main quantitative comparison

Table 3 summarizes the segmentation performance of SAM‑FT‑HN, SAM‑Med3D, and nnUNet across all evaluated H&N OARs using the DSC and HD95. Across several large and moderate-sized organs, including the brain, brainstem, mandible, and spinal cord, all three models showed relatively strong segmentation performance, with SAM-FT-HN and nnUNet generally achieving higher spatial overlap than SAM-Med3D. For representative large organs, SAM‑FT‑HN achieved Dice scores of 0.972 for the brain and 0.904 for the brainstem, compared with 0.963 and 0.891 for nnUNet and 0.889 and 0.795 for SAM-Med3D, respectively.

Across organ groups, SAM‑FT‑HN achieved a mean Dice of 0.814 for large organs, slightly higher than nnUNet (0.791) and substantially higher than SAM‑Med3D (0.609). For small organs, SAM‑FT‑HN achieved a mean Dice of 0.669, markedly improving over SAM‑Med3D (0.300), although nnUNet remained higher on average (0.720). Overall, SAM‑FT‑HN achieved a mean Dice of 0.770 (averaged across all organs), and 0.769 for nnUNet, while also showing a slightly lower mean HD95 (3.57 mm vs. 3.61 mm). These results show that SAM-FT-HN substantially improved upon the original SAM-Med3D baseline while achieving closely matched overall Dice values to nnUNet.

**Table 3:** Segmentation performance on the test set across large organs

Large organ	SAM-FT-HN Dice	SAM-FT-HN HD95 (mm)	SAM-Med3D Dice	SAM-Med3D HD95 (mm)	nnUNet Dice	nnUNet HD95 (mm)
Brain	0.972 ± 0.012	1.42 ± 0.38	0.889 ± 0.035	4.19 ± 1.23	0.963 ± 0.014	1.59 ± 0.42
Brainstem	0.904 ± 0.027	1.92 ± 0.75	0.795 ± 0.048	5.09 ± 1.45	0.891 ± 0.030	2.12 ± 0.81
Esophagus	0.728 ± 0.087	6.30 ± 1.72	0.569 ± 0.088	10.23 ± 3.54	0.747 ± 0.058	6.18 ± 1.63
L_brachialplexus	0.505 ± 0.090	8.36 ± 2.34	0.289 ± 0.122	21.42 ± 5.87	0.532 ± 0.095	8.24 ± 2.21
L_eye	0.926 ± 0.028	1.28 ± 0.35	0.648 ± 0.072	4.80 ± 1.32	0.880 ± 0.035	1.67 ± 0.42
L_parotid	0.845 ± 0.043	4.88 ± 1.42	0.643 ± 0.078	7.16 ± 2.01	0.825 ± 0.048	5.07 ± 1.47
L_submandible	0.839 ± 0.046	3.12 ± 0.92	0.580 ± 0.092	7.09 ± 2.50	0.813 ± 0.052	3.23 ± 1.03
Larynx	0.840 ± 0.052	2.91 ± 0.89	0.653 ± 0.076	9.27 ± 2.43	0.792 ± 0.058	2.97 ± 0.93
Lips	0.757 ± 0.068	6.28 ± 1.78	0.434 ± 0.145	9.57 ± 2.81	0.669 ± 0.072	6.58 ± 1.83
Mandible	0.904 ± 0.028	2.18 ± 0.62	0.754 ± 0.055	5.90 ± 1.52	0.887 ± 0.030	2.67 ± 0.60
Oralcavity	0.848 ± 0.054	4.28 ± 1.22	0.633 ± 0.080	8.60 ± 2.28	0.775 ± 0.056	5.06 ± 1.41
R_brachialplexus	0.522 ± 0.088	8.27 ± 2.24	0.297 ± 0.130	19.73 ± 5.65	0.550 ± 0.088	8.13 ± 2.18
R_eye	0.918 ± 0.031	1.35 ± 0.36	0.646 ± 0.076	5.16 ± 1.38	0.875 ± 0.032	1.69 ± 0.45
R_parotid	0.833 ± 0.048	5.02 ± 1.48	0.600 ± 0.089	7.53 ± 2.18	0.814 ± 0.052	5.24 ± 1.52
R_submandible	0.826 ± 0.051	3.05 ± 0.90	0.571 ± 0.098	7.24 ± 2.65	0.808 ± 0.054	3.29 ± 1.04
Spinalcord	0.851 ± 0.038	2.30 ± 0.57	0.744 ± 0.058	5.15 ± 1.82	0.827 ± 0.045	2.34 ± 0.65
Mean performance	0.814 ± 0.132	3.93 ± 2.37	0.609 ± 0.162	8.63 ± 5.01	0.791 ± 0.118	4.13 ± 2.26

Table 4 shows the per‑organ results for small organs specifically. For the optic chiasm, SAM‑FT‑HN improved Dice from 0.146 (SAM‑Med3D) to 0.618, a gain of 0.472. Similarly, the left optic nerve improved from 0.258 to 0.638 and the right optic nerve from 0.238 to 0.630. HD95 reductions were also substantial, with the chiasm decreasing from 10.07 mm to 4.02 mm and the optic nerves decreasing from 9.21 mm and 9.62 mm to 3.52 mm and 3.92 mm, respectively. Across all small organs, SAM‑FT‑HN improved mean Dice from 0.300 to 0.669 and reduced mean HD95 from 7.79 mm to 2.75 mm compared with SAM‑Med3D. These improvements support the effectiveness of the coarse‑to‑fine prompt‑guided approach for small‑organ segmentation, while nnUNet remained the strongest baseline in average small‑organ Dice.

**Table 4:** Segmentation performance on the test set across small organs

Small organ	SAM-FT-HN Dice	SAM-FT-HN HD95 (mm)	SAM-Med3D Dice	SAM-Med3D HD95 (mm)	nnUNet Dice	nnUNet HD95 (mm)
L_lens	0.735 ± 0.064	1.85 ± 0.52	0.395 ± 0.112	4.20 ± 1.15	0.765 ± 0.060	1.65 ± 0.48
R_lens	0.718 ± 0.068	1.98 ± 0.56	0.362 ± 0.108	4.50 ± 1.22	0.750 ± 0.062	1.76 ± 0.50
Chiasm	0.618 ± 0.090	4.02 ± 1.18	0.146 ± 0.135	10.07 ± 2.85	0.680 ± 0.082	3.54 ± 1.05
L_cochlea	0.675 ± 0.072	1.95 ± 0.56	0.361 ± 0.105	8.25 ± 2.32	0.746 ± 0.065	1.58 ± 0.45
R_cochlea	0.668 ± 0.078	2.00 ± 0.58	0.339 ± 0.107	8.65 ± 2.40	0.739 ± 0.067	1.63 ± 0.46
L_opticnerve	0.638 ± 0.085	3.52 ± 1.05	0.258 ± 0.118	9.21 ± 2.65	0.694 ± 0.078	3.07 ± 0.92
R_opticnerve	0.630 ± 0.087	3.92 ± 1.23	0.238 ± 0.115	9.62 ± 2.70	0.665 ± 0.082	3.77 ± 1.10
Mean Performance	0.669 ± 0.044	2.75 ± 1.01	0.300 ± 0.089	7.79 ± 2.42	0.720 ± 0.039	2.43 ± 0.99

Figure 2 presents boxplots of Dice distributions for small organs and large organs across the three evaluated models. Descriptively, SAM-FT-HN showed a substantially higher Dice distribution than SAM-Med3D for both small and large organs. Compared with nnUNet, SAM-FT-HN achieved a similar Dice distribution for large organs, whereas nnUNet remained higher for several small organs. These results indicate that SAM-FT-HN substantially improves performance relative to the baseline SAM-Med3D model while approaching nnUNet-level performance overall.

**Figure 2:**
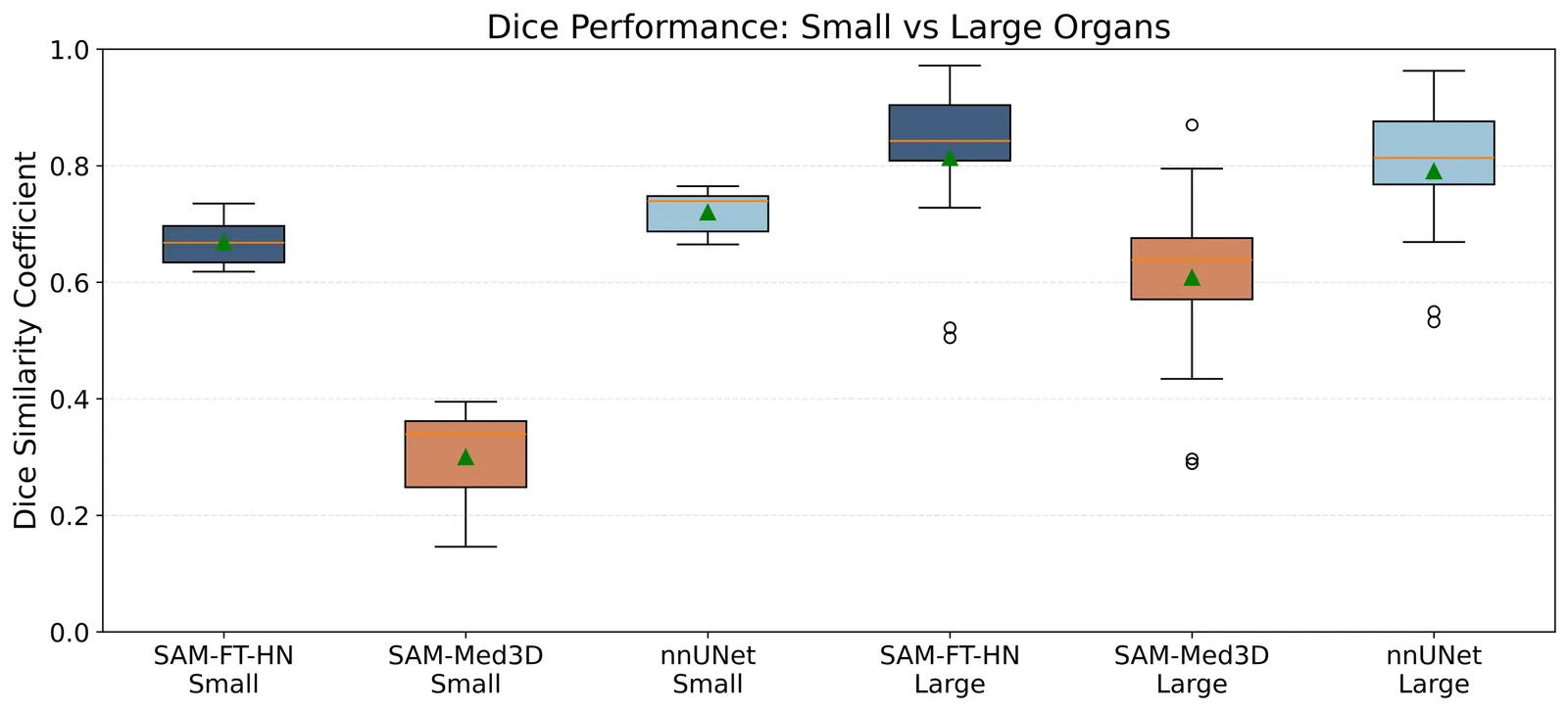
Boxplot of small and large organ segmentation Dice similarity performance SAM-Med3D: Segment Anything Model 3D medical variants, SAM-FT-HN: coarse-to-fine prompt-guided adaptation framework for the head and neck.

Per-organ small-organ performance

For very small but clinically critical structures including the optic nerves, lenses, cochleae, and optic chiasm (all < 1.5 cm³), SAM-FT-HN consistently improved segmentation accuracy compared with SAM-Med3D (Figure 3). The largest improvement was observed for the optic chiasm, where Dice increased from 0.146 to 0.618 and HD95 decreased from 10.07 mm to 4.02 mm. Across all small organs, SAM-FT-HN increased mean Dice from 0.300 to 0.669 and reduced mean HD95 by approximately 65% relative to SAM-Med3D. These improvements address common failure modes of the baseline model, including low soft-tissue contrast, limited voxel representation, and instability under fixed-threshold inference. By incorporating ROI-guided prompting and volume-consistent thresholding, SAM-FT-HN produced more stable and anatomically coherent small-organ segmentations, although nnUNet still achieved the highest average small-organ Dice.

**Figure 3:**
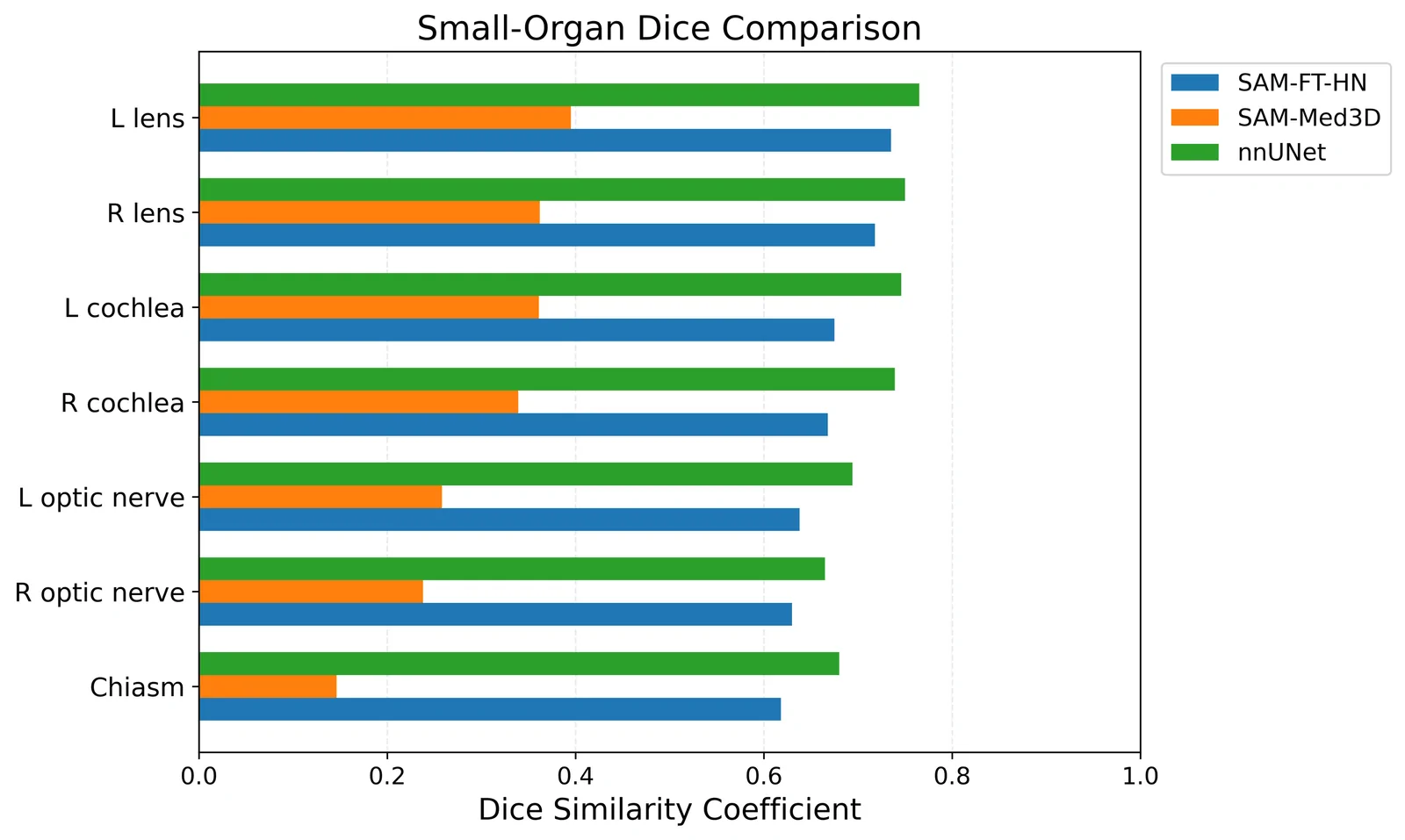
Dice score improvement across small structures: comparison between SAM-FT-HN, SAM-Med3D, and nnUNet SAM-Med3D: Segment Anything Model 3D medical variants, SAM-FT-HN: coarse-to-fine prompt-guided adaptation framework for the head and neck.

Ablation study

Table 5 presents the results of our ablation study, which progressively adds components to isolate their individual contributions. The original SAM-Med3D model achieved a mean Dice of 0.515 across all organs, with particularly poor performance on small organs (0.300). Fine-tuning SAM-Med3D on the H&N dataset improved overall Dice to 0.690, with small-organ Dice increasing to 0.485. Adding prompts from the coarse localization network further improved performance to 0.728 overall and 0.542 for small organs. The ROI-guided tiny-organ enhancement module provided an additional boost to 0.745 overall and 0.638 for small organs. Finally, adding organ-specific threshold calibration yielded the full method performance of 0.770 overall and 0.669 for small organs.

These results show that each component contributes meaningfully to the overall performance, with the largest gains coming from domain fine-tuning and the ROI-guided tiny-organ enhancement. Notably, the ROI-guided refinement was particularly beneficial for small organs, providing a +0.096 Dice improvement compared with the coarse-localization-prompted model, while having minimal effect on large-organ performance.

**Table 5:** Ablation study configurations and corresponding segmentation performance (Dice) on the test set

Variant	Overall Dice	Large-organ Dice	Small-organ Dice
SAM-Med3D	0.515	0.609	0.3
+ domain fine-tuning	0.69	0.77	0.485
+ coarse localization prompt	0.728	0.785	0.542
+ ROI-guided tiny-organ	0.745	0.789	0.638
Full method	0.77	0.814	0.669

Qualitative evaluation

Qualitative segmentation results are shown in Figure 4. The figure compares ground-truth contours with predictions from SAM-FT-HN, SAM-Med3D, and nnUNet across several head and neck organs. For large anatomical structures such as the brain, mandible, and parotid glands, SAM-FT-HN and nnUNet produced similar contour delineations with good spatial agreement relative to the ground truth. In contrast, SAM-Med3D occasionally showed minor boundary deviations. For smaller organs, clearer differences between the models were observed. In several examples, SAM-Med3D produced over- or under-segmentations or discontinuous segmentations for thin or low-contrast structures such as the optic nerves and cochleae. SAM-FT-HN generated more continuous contours with improved spatial alignment relative to the ground-truth boundaries. These qualitative observations are consistent with the quantitative improvements observed in overlap and boundary metrics.

**Figure 4:**
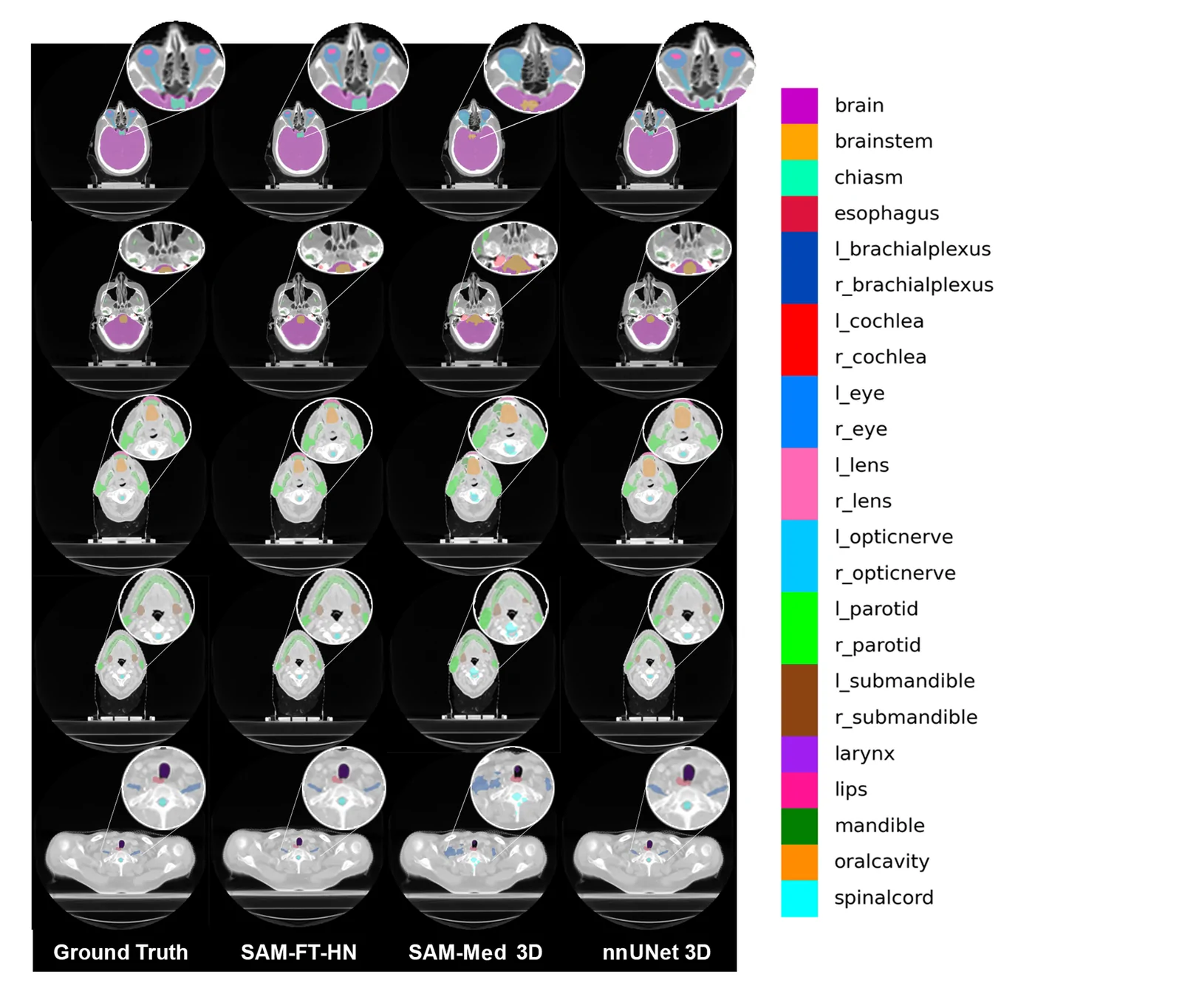
Qualitative comparison of ground-truth contours and predicted masks from SAM-FT-HN, SAM-Med3D, and nnUNet SAM-Med3D: Segment Anything Model 3D medical variants, SAM-FT-HN: coarse-to-fine prompt-guided adaptation framework for the head and neck. Image Credits: Individual panels were generated using Python 3.10 (Matplotlib), and the final composite figure was assembled using Microsoft PowerPoint on Windows 11 (Microsoft Corp., Redmond, WA, USA).

Statistical analysis

Patient-level statistical analysis was performed across the 42 test patients using the mean segmentation performance across the 23 H&N organs for each patient. Complete reference annotations for all 23 OARs were available for every patient in the test cohort. Because metrics were first averaged across organs within each patient and then summarized across patients, these patient-level results may differ slightly from the organ-wise averages reported in Table 3 and Table 4. For Dice, the overall Friedman test showed a significant difference among the three models ($\chi^2$(2) = 63.05, p < 0.001; Kendall's W = 0.751). Subsequent pairwise Wilcoxon signed-rank tests with Benjamini-Hochberg correction showed no statistically significant difference between SAM-FT-HN and nnUNet (W = 412, BH-adjusted p = 0.833). However, SAM-FT-HN achieved significantly higher Dice scores than SAM-Med3D (W = 861, BH-adjusted p < 0.001), and the same was observed for nnUNet compared with SAM-Med3D (W = 858, BH-adjusted p < 0.001). For HD95, the overall Friedman test also showed a significant difference among the three models ($\chi^2$(2) = 58.42, p < 0.001; Kendall's W = 0.696). Pairwise Wilcoxon signed-rank tests with Benjamini-Hochberg correction showed that SAM-FT-HN had significantly lower HD95 values than SAM-Med3D (W = 855, BH-adjusted p < 0.001) and nnUNet had significantly lower HD95 values than SAM-Med3D (W = 851, BH-adjusted p < 0.001). The difference between SAM-FT-HN and nnUNet was not statistically significant (W = 485, BH-adjusted p = 0.58). These results suggest that SAM-FT-HN and nnUNet performed similarly at the patient level, while both achieved significantly higher Dice scores and lower HD95 values than SAM-Med3D.

For the small-organ subgroup (42 patients), the overall Friedman test identified a significant difference among the three models for Dice ($\chi^2$(2) = 71.42, p < 0.001; Kendall's W = 0.850). Subsequent pairwise Wilcoxon signed-rank tests with Benjamini-Hochberg correction showed that both SAM-FT-HN and nnUNet significantly outperformed SAM-Med3D (W = 864, adjusted p < 0.001, and W = 859, adjusted p < 0.001, respectively). In contrast to the overall analysis, nnUNet also significantly outperformed SAM-FT-HN for small-organ Dice (W = 195, adjusted p < 0.001). A similar pattern was observed for HD95, where the overall Friedman test was significant ($\chi^2$(2) = 68.15, p < 0.001; Kendall's W = 0.811), and pairwise comparisons showed that SAM-FT-HN and nnUNet both had significantly lower HD95 values than SAM-Med3D (W = 858, adjusted p < 0.001, and W = 853, adjusted p < 0.001, respectively), while nnUNet also had significantly lower HD95 than SAM-FT-HN (W = 200, adjusted p < 0.001).

Figure 5 compares the mean accuracy, specificity, sensitivity, and precision of the three models across the evaluated H&N organs. Accuracy and specificity were high for all three models, which is expected because background voxels account for a large portion of volumetric medical images. The differences were more apparent for sensitivity and precision. SAM-FT-HN had higher sensitivity than both SAM-Med3D and nnUNet, indicating that it captured a larger proportion of the ground-truth organ voxels. Its precision was also higher than SAM-Med3D and only slightly lower than nnUNet. Overall, both SAM-FT-HN and nnUNet showed stronger voxel-level segmentation over SAM-Med3D.

**Figure 5:**
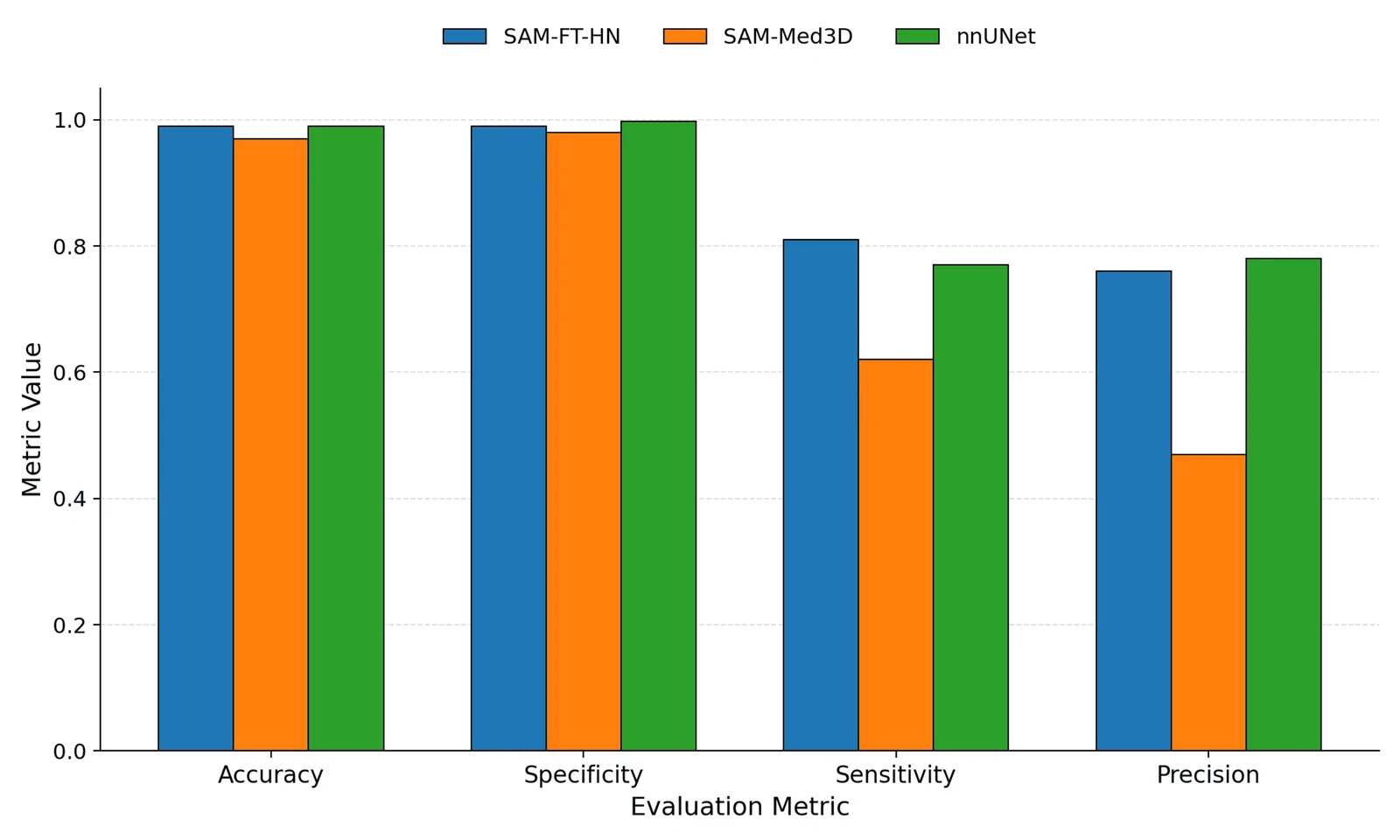
Average performance metrics across the three segmentation models

Figure 6 shows the relationship between organ volume and Dice score for SAM-FT-HN. Each point represents the mean volume and mean Dice score for one organ across the test cohort. When organ volume was plotted on a logarithmic scale, a positive association was observed (Pearson correlation: r = 0.647, t (21) = 3.89, p < 0.001; Spearman rank correlation: *ρ* = 0.708, p < 0.001). In general, larger organs tended to achieve higher Dice scores, while smaller structures exhibited greater variability and lower average performance. This pattern is consistent with the higher sensitivity of overlap‑based metrics to small boundary deviations in low‑volume organs.

**Figure 6:**
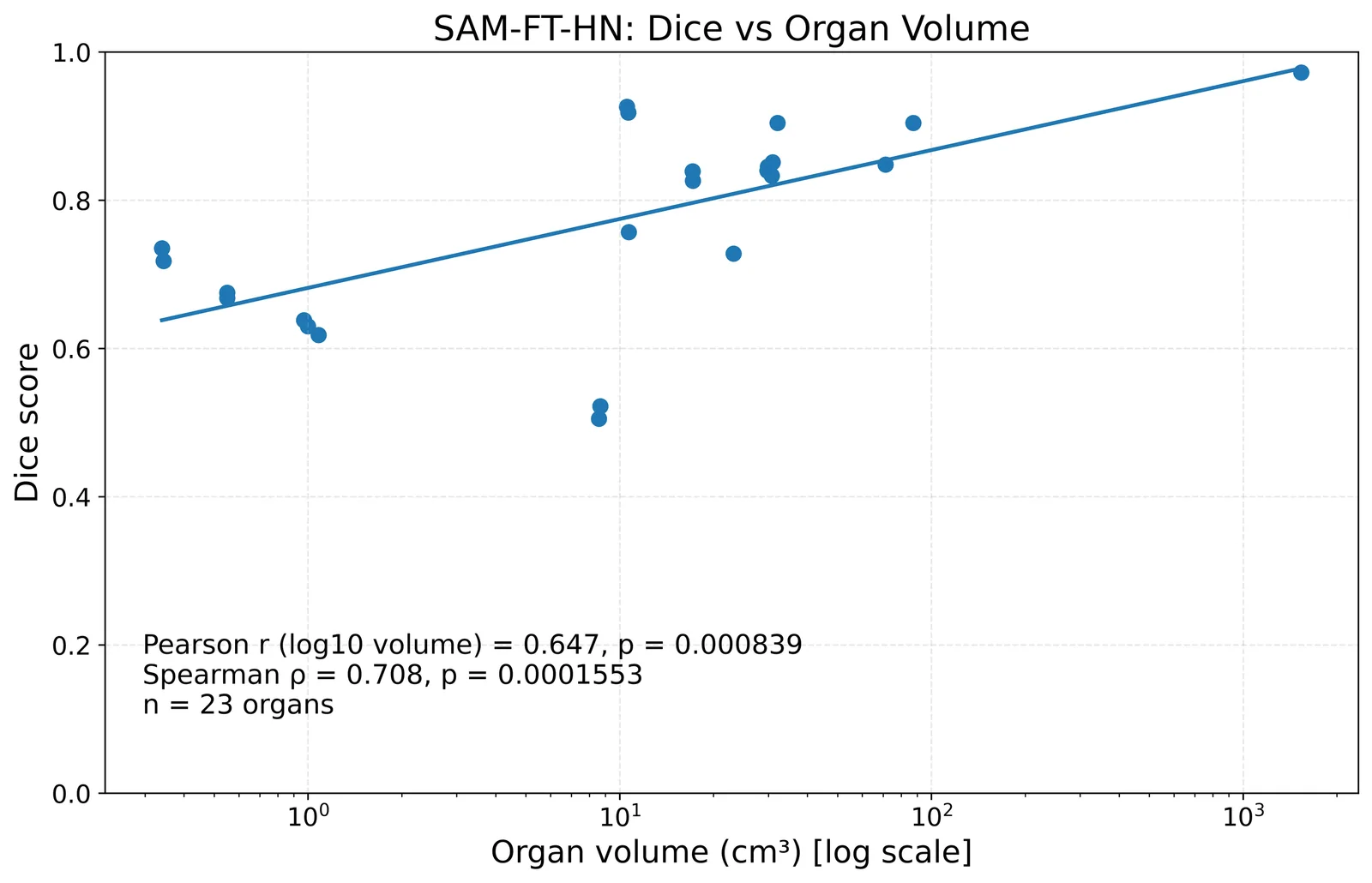
Relationship between organ volume (log scale) and Dice similarity coefficient for SAM-FT-HN Pearson correlation (r = 0.647, t (21) = 3.89, p < 0.001) and Spearman rank correlation (*ρ* = 0.708, p < 0.001) were used to assess the association. Each point represents the mean Dice score for a single organ. SAM-FT-HN: coarse-to-fine prompt-guided adaptation framework for the head and neck.

Failure analysis

We analyzed cases in which SAM-FT-HN showed poor segmentation performance (Dice < 0.5) to identify the primary sources of failure. A failure case was defined as a patient-organ instance with a Dice score below 0.5. Across the test set, 59 patient-organ instances met this criterion and were included in the failure analysis. Each instance was assigned to one of four mutually exclusive categories according to the earliest stage at which an error occurred in the segmentation pipeline: coarse localization error, prompt placement errors, segmentation under/over-segmentation, or empty segmentation (Table 6). For cases in which multiple potential causes were identified, the case was assigned to the earliest applicable failure category to avoid double counting.

**Table 6:** Failure mode distribution for SAM-FT-HN on cases with Dice < 0.5 Percentages reflect the proportion of all poor-performing cases. SAM-FT-HN: coarse-to-fine prompt-guided adaptation framework for the head and neck.

Failure mode	Definition	Proportion
Coarse localization error	Centroid error >5 mm or coarse mask coverage <90%	47.5% (28/59)
Prompt placement error	Localization criteria were satisfied, but the automatically generated prompt was located outside the ground-truth organ mask	20.3% (12/59)
Under-/over-segmentation	Localization and prompt placement were adequate, but the final mask substantially under- or over-segmented the target structure	18.6 % (11/59)
Empty segmentation	Localization and prompt generation were completed, but SAM-FT-HN produced no foreground voxels in the final segmentation mask	13.6% (8/59)
Total		100.0% (59/59)

Among the 59 patient-organ instances with poor segmentation performance (Dice < 0.5), coarse localization error was the most common failure mode, accounting for 47.5% (28/59) of cases. Prompt placement errors represented the second most frequent source of failure, occurring in 20.3% (12/59) of cases despite satisfactory coarse localization. Under- or over-segmentation accounted for 18.6% (11/59) of failures, indicating that inaccurate final boundaries could still occur when both localization and prompt placement were adequate. Empty segmentations were the least frequent failure mode, accounting for 13.6% (8/59) of cases, in which localization and prompt generation were successfully completed but the model produced no foreground voxels. Overall, these findings indicate that errors arising during the coarse localization stage were the dominant source of poor segmentation performance.

## Discussion

The results of this study show that SAM-FT-HN provides reliable segmentation performance across a wide range of H&N organs while substantially improving segmentation of anatomically challenging regions compared with the baseline SAM-Med3D model. Our findings indicate that the proposed coarse-to-fine prompt-guided framework, combined with ROI-guided refinement and organ-specific threshold calibration, effectively addresses several limitations of current foundation models for H&N radiotherapy segmentation.

One of the key contributions of our work is the automatic prompt generation strategy. Many previous studies using SAM for medical image segmentation have relied on manual or ground-truth-derived prompts [19,21,22], which limits their clinical relevance. By introducing a lightweight coarse localization network that automatically generates organ-specific prompts and ROIs, we have made the framework fully automatic in the primary inference setting while still preserving the flexibility of prompt-based interaction for potential user refinement. Our results show that the coarse localization network provides sufficient spatial accuracy (mean centroid error of 3.7 mm across all organs) to support downstream segmentation, with mean coarse mask coverage of 96.6% for large organs and 91.8% for small organs, ensuring that the vast majority of cases receive reliable initial prompts without any manual intervention. This addresses a critical gap in the literature: how to make promptable foundation models operational in real-world clinical settings without requiring user input or ground-truth guidance, while retaining the option for interactive refinement when needed.

The ablation analysis further showed the contribution of anatomically informed prompting. Adding coarse-localization-derived prompts after domain fine-tuning increased the overall Dice from 0.690 to 0.728 (+0.038). The improvement suggests that domain adaptation alone does not fully resolve the prompt sensitivity of SAM-based segmentation and that providing prompts derived from approximate anatomical localization can further improve segmentation performance. This finding supports the use of automated anatomical localization as an intermediate step between image input and prompt-based segmentation.

The tiny-organ ROI refinement module had a particularly strong effect on small-organ performance. In the ablation study, adding this component increased the mean small-organ Dice from 0.542 to 0.638 (+0.096). This difference is consistent with the intended purpose of the refinement strategy: restricting processing to a localized region reduces the relative contribution of surrounding background and increases the representation of small structures within the model input. Such localization is particularly important for structures such as the optic nerves, cochleae, lenses, and optic chiasm, where small absolute boundary deviations can result in substantial reductions in overlap-based metrics. The organ-specific ROI radii, determined on the validation set, were critical for this module's success. Using a radius that was too small risked excluding part of the organ, while a radius that was too large reintroduced background interference. Our validation-based optimization of this parameter ensured that the ROI was appropriate for each organ's typical size while accommodating anatomical variability.

Organ-specific threshold calibration provided an additional improvement in segmentation performance. Replacing a single fixed probability threshold with thresholds selected for individual organs on the validation set increased the overall Dice from 0.745 to 0.770 in the ablation study. Because H&N structures differ substantially in size, contrast, and boundary characteristics, a single threshold may not provide optimal binarization across all organs. The observed improvement suggests that validation-based, organ-specific calibration is a relatively simple but useful post-processing strategy for improving the consistency of predicted masks. Importantly, threshold selection was performed using the validation set rather than the test set to avoid optimization on the final evaluation data.

Our comparison with nnUNet provides important context for interpreting our results. nnUNet remains a very strong baseline for medical image segmentation [11]. SAM-FT-HN achieved higher Dice than nnUNet for several large organs, including the brain, brainstem, eyes, parotid glands, mandible, oral cavity, and spinal cord, whereas nnUNet maintained an advantage for several small organs. For example, nnUNet achieved Dice scores of 0.680 and 0.765 for the optic chiasm and left lens, respectively, compared with 0.618 and 0.735 for SAM-FT-HN.

However, nnUNet has several limitations that SAM-FT-HN addresses. First, nnUNet produces a single feed-forward prediction without opportunities for interactive refinement. If the initial segmentation is incorrect, as may occur with atypical anatomy or poor image quality, nnUNet does not provide prompt-conditioned interactive refinement. Therefore, erroneous contours typically require external manual editing or an additional correction workflow. In contrast, the prompt-based architecture of SAM-FT-HN provides the potential for interactive refinement when additional user guidance is available. Second, nnUNet is task-specific and requires retraining when new organs or modalities are introduced. Foundation-model-based frameworks such as SAM-FT-HN are more flexible and can potentially be adapted to new tasks with less training data [17].

The gap between SAM-FT-HN and nnUNet for small organs suggests that there is still room for improvement in SAM-based models' ability to handle structures with very limited voxel representation. One reason is that nnUNet's patch-based training strategy provides a form of scale specialization the network learns to work with patches of different sizes, which helps it capture fine details at appropriate scales. SAM, with its transformer-based global attention, may not be as efficient at localizing fine-scale features in small structures. Future work could explore hybrid approaches that combine SAM's global context modeling with more localized processing for small structures.

There are several limitations of this study. First, our dataset came from a single institution, which limits generalizability. Although the dataset included 211 patients treated over several years, external validation on data from other institutions with different imaging protocols and patient populations would be valuable to assess the model's robustness. Second, our coarse localization network, while sufficient for this study, could be improved. The 2.2% failure rate, particularly for challenging structures like the optic chiasm, indicates opportunities for more robust localization. Future work could explore using larger or more sophisticated localization networks, or incorporating anatomical priors to improve localization of small structures. Third, the current framework is limited to CT images and does not handle multimodal inputs. In clinical practice, H&N radiotherapy planning often uses additional imaging modalities such as MRI or PET to improve target and OAR delineation. Extending SAM-FT-HN to handle multimodal inputs could further improve segmentation accuracy, particularly in regions where CT contrast is poor.

Another important limitation of the proposed framework is the additional architectural and computational complexity introduced by combining a coarse 3D localization network with a foundation-model segmentation stage. Although this design enables fully automatic anatomy-aware prompt generation, errors in coarse localization can propagate to the downstream segmentation. Moreover, nnUNet achieved higher mean Dice performance for small organs. nnUNet showed the lowest computational requirements, with an inference time of 21.8 s per volume and a GPU memory usage of 18.7 GB during training. In comparison, SAM-Med3D required 46.3 s per volume and 41.2 GB of GPU memory, while SAM-FT-HN required 48.5 s per volume and 44.6 GB. However, our findings show the feasibility of adapting a prompt-based foundation model to a fully automatic H&N segmentation workflow while substantially improving upon the original SAM-Med3D. The proposed design eliminates the need for manual prompt placement by generating anatomy-aware prompts from coarse localization, while retaining the prompt-conditioned segmentation capability of SAM-Med3D. Future work will focus on improving the robustness of the coarse localization stage and reducing the computational overhead of the two-stage framework. The present study represents a technical segmentation validation rather than a clinical validation study. Although the proposed framework demonstrated promising segmentation performance, its clinical utility was not directly evaluated through physician workflow integration, dosimetric analysis, or prospective clinical assessment. Future work should focus on incorporating physician review and dosimetric evaluation to determine the clinical value of the proposed framework.

Future research directions include exploring text-based prompts for segmentation guidance. With the emergence of vision-language models such as Contrastive Language-Image Pre-training (CLIP) [30], it is becoming possible to use semantic descriptions to guide segmentation, for example, prompting the model to segment "the optic nerve" rather than pointing at it. This could further simplify clinical interaction and reduce the cognitive load on clinicians. Additionally, combining SAM with large language models could enable the system to interpret clinical intent and generate or refine prompts automatically, potentially improving segmentation accuracy and workflow integration. We also plan to explore adaptation strategies for low-resource settings. Foundation models like SAM are trained on massive datasets, but their fine-tuning often still requires substantial labeled data. Techniques such as few-shot learning or test-time adaptation could reduce the labeling burden and make these models more accessible for clinical implementation. Future work will also extend the evaluation beyond geometric segmentation metrics by incorporating DVH-based dosimetric analysis.

This work contributes to the growing body of research on adapting foundation models to medical image segmentation. Our results show that domain-specific fine-tuning and task-specific enhancements, particularly automatic prompt generation and coarse-to-fine localization, can substantially improve SAM-based performance on challenging small structures. In practice, SAM‑FT‑HN complements nnUNet by offering strong segmentation accuracy with a different design trade‑off, while its automatic prompting mechanism reduces reliance on manual or ground‑truth‑derived prompts. These findings may be relevant to other segmentation tasks where small, poorly contrasted targets pose similar difficulties. The finding that SAM-FT-HN achieves closely matched Dice performance with nnUNet for large organs while offering prompt-guided refinement suggests that foundation models may complement traditional CNN-based approaches in clinical practice in the future. Rather than choosing one approach over the other, future clinical systems might combine them, using nnUNet or similar models for fully automated segmentation of well-characterized structures, and SAM-based models for interactive refinement of challenging cases or for new tasks where labeled data are scarce.

## Conclusions

In this study, we proposed SAM-FT-HN, an anatomy-aware, coarse-to-fine framework for fully automatic H&N OAR segmentation. By integrating automatic prompt generation through coarse localization, ROI-guided refinement for small organs, and organ-specific threshold calibration, the proposed method substantially improved the original SAM-Med3D model. Overall Dice performance was closely matched between SAM-FT-HN and nnUNet, although nnUNet achieved a higher mean Dice for the small-organ subgroup. These results suggest that promptable foundation models, when appropriately adapted with task-specific strategies, can effectively address complex radiotherapy segmentation tasks. Although challenges remain for the most anatomically complex structures, our findings present the potential of anatomy-aware, promptable foundation models for H&N radiotherapy segmentation and provide a foundation for future external and clinical validation.
